# Bilateral Foveal Damage Induced by Indirect Picosecond Nd:YAG Laser Exposure: A Case Report

**DOI:** 10.1155/crop/6664488

**Published:** 2025-02-07

**Authors:** Takahiro Miyake, Naoki Kimura, Fumi Gomi

**Affiliations:** Department of Ophthalmology, Hyogo Medical University, Nishinomiya-shi, Hyogo, Japan

## Abstract

**Introduction:** Accidental retinal injuries caused by lasers without appropriate eye protection are not rare; most cases are unilateral. We report the case of a medical nurse who sustained bilateral foveal damage through indirect exposure to a picosecond dermal laser.

**Case Presentation:** A 23-year-old nurse working in a cosmetic surgery clinic was using a picosecond KTP/Nd:YAG laser for tattoo removal. Because the procedure was complicated, she neglected the use of protective eyewear and experienced dazzle. Thirty minutes after starting the procedure, she developed central scotomas in both eyes. We examined her eyes the next day. Ophthalmologic examination revealed best-corrected decimal visual acuity (BCVA) of 0.6 in the right eye and 0.3 in the left eye. Spectral domain–optical coherence tomography showed a hyperreflective inner retinal layer with a lamellar defect and focal outer retinal detachment in the right eye; the left eye exhibited intra- and subretinal foveal hemorrhages. Injections of sub-Tenon's triamcinolone acetonide (12 mg/0.3 mL) in the right eye and intravitreal tissue plasminogen activator (30 *μ*g/0.05 mL) in the left eye were administered on the same day. Two weeks later, a full-thickness macular hole (FTMH) was identified in the right eye; pars plana vitrectomy was required 6 weeks after initial presentation. Because the FTMH failed to close, a second procedure was performed 2 months later. One year after initial presentation, BCVA in the right eye had improved to 0.4. Although the FTMH remained closed, an outer retinal layer defect persisted. In the left eye, foveal hemorrhage resolved within 1 month of initial presentation. At the 1-year follow-up, BCVA in the left eye was 0.4; outer retinal layer disruption was evident at the central fovea.

**Conclusions:** Continuous Nd:YAG laser exposure during cosmetic procedures likely caused the bilateral foveal damage observed in this case. All individuals using lasers must be aware of the importance of protective goggles.

## 1. Introduction

Picosecond lasers are widely used by dermatologists for tattoo removal and the treatment of benign pigmented lesions, pigmentary disorders, acne scars, and skin photoaging [1]. Lasers are also extensively utilized in scientific research and industrial applications [2]. Laser exposure carries substantial risks to ocular health, and protective eyewear is essential [3], particularly when using short-wavelength pulsed lasers [4]. Nonetheless, accidental retinal injuries in the absence of adequate eye protection have been reported [2, 5–7]. We report the case of a nurse who sustained bilateral foveal damage through indirect exposure to a picosecond dermal laser.

## 2. Case Presentation

**Figure 1:**
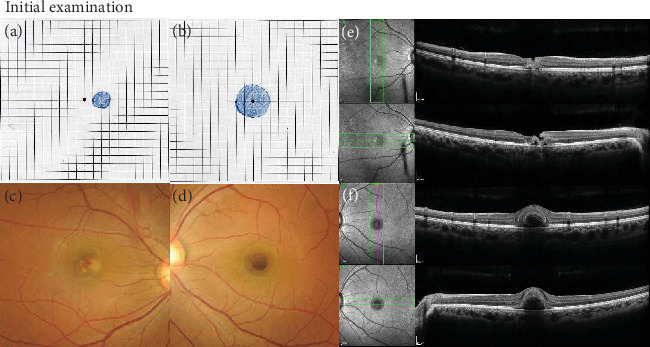
Baseline findings. (a) Initial Amsler grid testing revealed a paracentral scotoma in the right eye and (b) a larger central scotoma in the left eye. (c) Fundus examination of the right eye showed faint vitreous hemorrhage and several white spots with retinal hemorrhage at the macula; (d) the left eye displayed a thick hemorrhage at the central fovea. (e) OCT of the right eye demonstrated a hyperreflective inner retinal layer with a lamellar defect and focal detachment of the outer retinal layer. (f) OCT of the left eye revealed intra- and subretinal hemorrhages at the fovea.

A 23-year-old nurse was operating a picosecond KTP/Nd:YAG laser for tattoo removal in a cosmetic surgery clinic without wearing protective goggles due to the complexity of the procedure. During the operation, she experienced dazzle via reflected light. Thirty minutes after procedure completion, she suddenly developed a central scotoma in both eyes. The next day, she was referred to our hospital. Her best-corrected visual acuity (BCVA) was 0.6 in the right eye and 0.3 in the left eye. Amsler grid testing revealed a paracentral scotoma in the right eye (Figure 1(a)) and a larger central scotoma in the left eye (Figure 1(b)). Fundus examination of the right eye showed faint vitreous hemorrhage and several white spots with retinal hemorrhage at the macula (Figure 1(c)); the left eye displayed dense hemorrhage at the central fovea (Figure 1(d)). Spectral domain–optical coherence tomography (OCT) showed a juxtafoveal full-thickness hyperreflectivity with lamellar defect and focal subretinal detachment just on the nasal side of the fovea (Figure 1(e)). In the left eye, intra- and subretinal hemorrhages at the fovea were observed (Figure 1(f)). On the same day, the patient received injections of sub-Tenon's triamcinolone acetonide (STTA) (12 mg/0.3 mL) in the right eye and intravitreal tissue plasminogen activator (t-PA) (30 *μ*g/0.05 mL) in the left eye.

**Figure 2:**
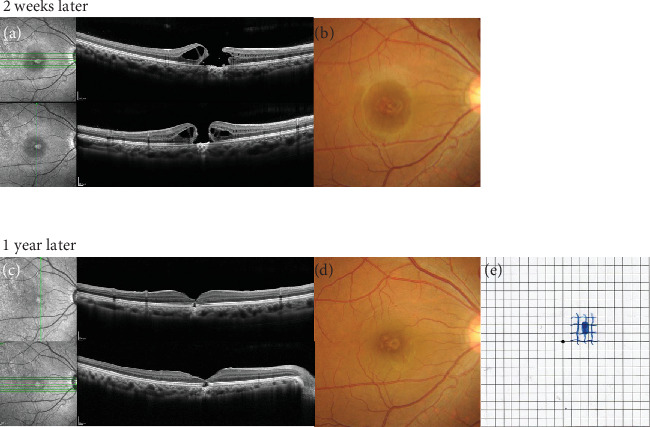
Time course in the right eye. Two weeks after the initial examination, (a) OCT showed an FTMH nasal to the fovea; (b) fundus examination revealed several white spots and the FTMH at the macula. At the 1-year follow-up after two PPV procedures, (c) OCT confirmed closure of the FTMH, although a defect in the outer retinal layer persisted; (d) fundus examination showed residual white spots at the fovea; and (e) Amsler grid testing indicated distorted vision adjacent to the central scotoma.

In the right eye, a full-thickness macular hole (FTMH) was detected on the nasal side of the fovea 2 weeks later on OCT (Figure 2(a)); BCVA was 0.5. Fundus examination revealed several white spots and an FTMH at the macula (Figure 2(b)). Spontaneous closure of the FTMH was expected, but it remained open after 1 month; thus, we performed pars plana vitrectomy (PPV) using a 27-gauge trocar system. During the procedure, posterior vitreous detachment was induced artificially; this was followed by triamcinolone acetonide-assisted vitrectomy. The inner limiting membrane (ILM) was stained with Brilliant Blue G dye for visualization. The ILM within the fovea and the temporal half of the ILM were peeled; the temporal half of the ILM was inverted over the macular hole (MH) using the inverted ILM flap technique. Fluid-air exchange was performed, and 20% sulfur hexafluoride gas was used as a tamponade. Postoperative OCT showed crosslinking of the ILM but no closure of the FTMH. Two months after the initial surgery, a second PPV was performed with additional ILM peeling and 5% sulfur hexafluoride gas tamponade. This second operation resulted in the successful closure of the FTMH. One year after the initial examination, BCVA in the right eye had improved to 0.4. The FTMH remained closed, although a defect in the outer retinal layer persisted (Figure 2(c)). On fundus examination, white spots at the fovea remained visible (Figure 2(d)). Amsler grid testing showed a reduction in the distorted vision. The paracentral scotoma decreased in size and did not involve the fovea (Figure 2(e)).

**Figure 3:**
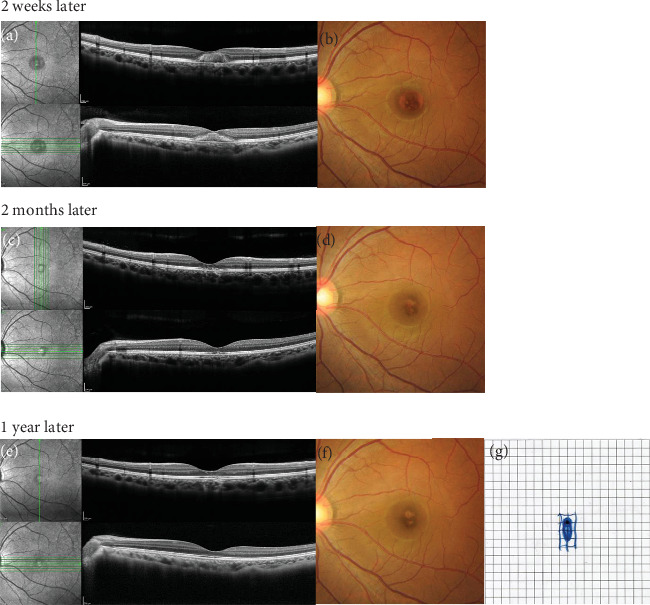
Time course in the left eye. Two weeks after treatment, (a) OCT showed a reduction in the foveal hemorrhage; (b) fundus examination corroborated the decrease in foveal hemorrhage. Two months after the initial examination, (c) OCT showed resolution of the subretinal hemorrhages and identified a defect in the ellipsoid zone at the central fovea; (d) fundus examination confirmed the disappearance of intra- and subretinal hemorrhages at the fovea. One year after the initial examination, (e) OCT showed a persistent defect in the ellipsoid zone at the central fovea without residual subretinal hemorrhages; (f) fundus examination confirmed the absence of intra- and subretinal hemorrhages at the fovea; and (g) Amsler grid testing showed a decrease in the size of the central scotoma.

In the left eye, a decrease in foveal hemorrhage was confirmed within 2 weeks after treatment on OCT (Figure 3(a)) and fundus examination (Figure 3(b)). One month after the initial examination, BCVA in the left eye had improved to 0.7, and the foveal hemorrhage had completely resolved. The patient also reported a reduction in the size of the central scotoma. Two months after the initial examination, OCT revealed the resolution of subretinal hemorrhages but identified a defect in the ellipsoid zone at the central fovea (Figure 3(c)). Fundus examination showed that intra- and subretinal hemorrhages at the fovea had resolved (Figure 3(d)). One year after the initial examination, BCVA in the left eye was 0.4. OCT confirmed the presence of an ellipsoid zone defect at the central fovea; no subretinal hemorrhages remained (Figure 3(e)). Fundus examination revealed no residual intra- or subretinal hemorrhages at the fovea (Figure 3(f)). Amsler grid testing indicated that the size of the central scotoma had further decreased (Figure 3(g)).

## 3. Discussion

The laser-induced damage is divided into photochemical, thermal, and thermomechanical damage mechanisms depending on the power density, pulse duration, and wavelength [8]. The laser wavelength from 350 to 1500 nm can be absorbed by human and rabbit ocular media, retinal pigment epithelium, and choroid [9], and the longer laser wavelengths of Nd:YAG (1064 nm) can achieve greater transmission than commonly used green (495–570 nm) and yellow (570–590 nm) lasers [10]. Retinal laser injuries result from tissue disruption caused by short bursts of high-power laser pulses. Experiments in monkeys have demonstrated the highly localized tissue damage induced by picosecond Nd:YAG lasers [4]. In the current case, picosecond Nd:YAG laser (the pulse duration, 339 ps) damaged both her eyes thermo-mechanically.

Commiskey, Heisel, and Paulus summarized laser-induced retinal injuries in a 2019 review article [11]. With the increasing use of Nd:YAG lasers in dermatology and cosmetic surgery, multiple cases of retinal injuries in these contexts have been reported [1, 2, 6, 12–15]. In the present case, the nurse operated an Nd:YAG laser for a cosmetic procedure and reported experiencing visual scotomas in both eyes approximately 30 min after starting the procedure. It is likely that cumulative damage from reflected laser light caused bilateral foveal damage.

In the right eye, juxtafoveal full-thickness retinal hyperreflectivity and disruption of the photoreceptor outer layer were initially observed. An injection of STTA was administered to mitigate the injury by decreasing the laser-induced cellular inflammatory response in the retina and retinal pigment epithelium [16]. Despite this intervention, an FTMH developed. Systemic corticosteroids have been used to treat laser-induced maculopathy with the aim of accelerating recovery. Hossein et al. [17] reported improvement in laser-induced foveal changes on OCT in a patient treated with high-dose systemic steroids. According to their report, after 1 week of treatment, the hyperreflective band at the fovea resolved, although residual disruption of the outer retinal layer at the fovea remained unchanged. The effectiveness of steroids in such cases is inconclusive and likely depends on the extent of the damage.

Kuwayama et al. [5] and Jbara Tiuseco and Azar [14] described patients who developed MHs immediately after cosmetic laser injuries, attributed to retinal layer destruction. According to previous reports, laser-induced MHs often enlarge over time, leading to diminished visual acuity [18]. Alsulaiman et al. reported spontaneous closure of only one out of 17 MH cases caused by a blue laser. This particular MH, with a minimum diameter of 168 *μ*m, was the smallest reported [19]. In the present case, when the FTMH was identified 2 weeks after the initial examination, its minimum diameter was 270 *μ*m. Based on this size, we suspected that spontaneous closure was unlikely. As expected, the MH had enlarged 2 weeks later; we thus performed PPV.

Qi et al. reported 11 cases of laser-induced MHs that were successfully closed using PPV with a peeled ILM flap [20]. In our patient, two operations were required to achieve MH closure; wider ILM peeling was performed during the second procedure. Early vitrectomy with more extensive ILM peeling is recommended to improve the likelihood of successful MH closure. In the report by Qi et al. [20], OCT findings after MH closure revealed varying degrees of outer retinal damage, including disruption of the external limiting membrane, outer photoreceptor ellipsoid and interdigitation bands, subfoveal hyperreflectivity, and focal choroidal depression. The latter two findings suggest deeper damage involving the retinal pigment epithelium and choriocapillaris. In the present case, all of these abnormalities were observed on OCT. These structural changes may explain the dark spot observed in the patient's right eye.

In the left eye, foveal hemorrhage was detected in the intra- and subretinal regions on OCT, likely caused by ruptured retinal and/or choroidal capillaries. Considering that the primary symptom in the left eye was a central scotoma, t-PA was injected intravitreally to promote faster resolution of the hemorrhage. Prolonged subretinal hemorrhages are known to cause permanent photoreceptor damage; t-PA injections are frequently used to dissolve intra- and subretinal hemorrhages, including those associated with traumatic submacular hemorrhage [21]. Kimura et al. [22] reported complete liquefaction of acute-onset subretinal blood 12–36 h after intravitreal t-PA injection. In the present case, we administered t-PA for the subfoveal hemorrhage and observed a rapid reduction of hemorrhage on OCT. Although submacular hemorrhage itself can result in MH formation [23], no MH developed in our patient's left eye. However, t-PA may have retinal toxicity, particularly at higher doses [24]; caution is warranted when using t-PA in eyes with laser-induced retinal damage. At the 1-year follow-up, the patient continued to report a central scotoma in the left eye, and OCT revealed a focal defect in the ellipsoid zone with adjacent irregularity. Further studies are needed to confirm the benefits of t-PA for treating laser-induced hemorrhages.

Our patient did not wear protective goggles during a complex procedure involving the Nd:YAG laser, despite receiving instructions to wear them. Laser exposure can lead to visual impairment in both eyes. As repeatedly emphasized, all individuals using Nd:YAG lasers must be aware of the risk of visual damage and must strictly adhere to the use of protective goggles.

## Data Availability

The data that support the findings of this study are available from the corresponding author upon reasonable request.
